# Deep learning, computer-aided radiography reading for tuberculosis: a diagnostic accuracy study from a tertiary hospital in India

**DOI:** 10.1038/s41598-019-56589-3

**Published:** 2020-01-14

**Authors:** Madlen Nash, Rajagopal Kadavigere, Jasbon Andrade, Cynthia Amrutha Sukumar, Kiran Chawla, Vishnu Prasad Shenoy, Tripti Pande, Sophie Huddart, Madhukar Pai, Kavitha Saravu

**Affiliations:** 1 Department of Epidemiology, Biostatistics and Occupational Health, McGill University, Montreal, Canada; 2 McGill International TB Centre, McGill University, Montreal, Canada; 3 Department of Radiodiagnosis, Kasturba Medical College, Manipal, Manipal Academy of Higher Education, Manipal, India; 4 Department of Medicine, Kasturba Medical College, Manipal, Manipal Academy of Higher Education, Manipal, India; 5 Department of Microbiology, Kasturba Medical College, Manipal, Manipal Academy of Higher Education, Manipal, India; 6 Department of Infectious Diseases, Kasturba Medical College, Manipal, Manipal Academy of Higher Education, Manipal, India; 7 Manipal McGill Program for Infectious Diseases, Manipal Centre for Infectious Diseases, Prasanna School of Public Health, Manipal Academy of Higher Education, Manipal, India

**Keywords:** Tuberculosis, Pathology, Radiography

## Abstract

In general, chest radiographs (CXR) have high sensitivity and moderate specificity for active pulmonary tuberculosis (PTB) screening when interpreted by human readers. However, they are challenging to scale due to hardware costs and the dearth of professionals available to interpret CXR in low-resource, high PTB burden settings. Recently, several computer-aided detection (CAD) programs have been developed to facilitate automated CXR interpretation. We conducted a retrospective case-control study to assess the diagnostic accuracy of a CAD software (*qXR*, Qure.ai, Mumbai, India) using microbiologically-confirmed PTB as the reference standard. To assess overall accuracy of *qXR*, receiver operating characteristic (ROC) analysis was used to determine the area under the curve (AUC), along with 95% confidence intervals (CI). Kappa coefficients, and associated 95% CI, were used to investigate inter-rater reliability of the radiologists for detection of specific chest abnormalities. In total, 317 cases and 612 controls were included in the analysis. The AUC for *qXR* for the detection of microbiologically-confirmed PTB was 0.81 (95% CI: 0.78, 0.84). Using the threshold that maximized sensitivity and specificity of *qXR* simultaneously, the software achieved a sensitivity and specificity of 71% (95% CI: 66%, 76%) and 80% (95% CI: 77%, 83%), respectively. The sensitivity and specificity of radiologists for the detection of microbiologically-confirmed PTB was 56% (95% CI: 50%, 62%) and 80% (95% CI: 77%, 83%), respectively. For detection of key PTB-related abnormalities ‘pleural effusion’ and ‘cavity’, *qXR* achieved an AUC of 0.94 (95% CI: 0.92, 0.96) and 0.84 (95% CI: 0.82, 0.87), respectively. For the other abnormalities, the AUC ranged from 0.75 (95% CI: 0.70, 0.80) to 0.94 (95% CI: 0.91, 0.96). The controls had a high prevalence of other lung diseases which can cause radiological manifestations similar to PTB (e.g., 26% had pneumonia, 15% had lung malignancy, etc.). In a tertiary hospital in India, *qXR* demonstrated moderate sensitivity and specificity for the detection of PTB. There is likely a larger role for CAD software as a triage test for PTB at the primary care level in settings where access to radiologists in limited. Larger prospective studies that can better assess heterogeneity in important subgroups are needed.

## Introduction

Tuberculosis (TB) is the world’s leading infectious disease killer. Early and accurate detection of TB is essential to achieve global control of the disease. However, many high-burden countries continue to have large gaps in TB diagnosis. India has the world’s largest TB burden and accounts for over one quarter of the 3.8 million ‘missing cases’ which go undiagnosed each year^1^. This gap is largely due to the lack of accurate, rapid and cost-effective tools for TB screening and diagnosis^2^.

The use of chest radiography (CXR) for the diagnosis of pulmonary TB (PTB) dates back over a century^3^. However, the utility of CXR is limited by modest specificity, high hardware costs, and poor inter-rater reliability^4–6^. In addition, many high burden countries, including India, suffer from a lack of qualified radiologists to interpret CXR^7^. Now with the advent of digital CXR, there is renewed interest in using CXR interpreted by computer-aided detection (CAD) software programs for PTB detection^3^. These programs have the potential to overcome many of the barriers to CXR-based screening and triage by standardizing and automating CXR interpretation. However, due to the limited and heterogeneous data available, the World Health Organization (WHO) has yet to initiate the guideline development process for the use of CAD for PTB detection^8^.

In 2018, a new commercial CAD software with the capacity for PTB detection, *qXR* (Qure.ai, Mumbai, India), received CE-certification^9^. According to the company, the software is compatible with all radiology information technology (IT) system, integrates easily with radiology workflow and can processes one CXR in 10 milliseconds. However, details on the cost of the software are not publicly available.

The objective of this study was to assess the diagnostic accuracy of *qXR* Version 2 for triage of presumptive PTB patients in tertiary hospital in India. We assessed the software’s performance for detection of PTB using microbiological confirmation as a reference standard and the software’s performance for detection of specific chest abnormalities (e.g., cavities) using radiologist’s readings as a reference standard.

## Methods

### Study setting

This study was conducted at Kasturba Hospital, Manipal, a large private tertiary health care facility. The study base was individuals who presented to Kasturba Hospital with respiratory symptoms for which they received a CXR and underwent confirmatory microbiological testing for PTB during January 1^st^, 2017 – December 31^st^, 2017. Both the cases and controls were selected from this study base.

### Sample size

Based on prior knowledge of the approximate annual number of PTB cases at Kasturba Hospital, we estimated between 300–400 cases and 600–800 controls would present over the year. Assuming the area under the curve (AUC) of *qXR* for detection of microbiologically-confirmed PTB to be non-inferior to that of CAD4TB Version 6 (the other commercially available CAD software for PTB), we needed 323 cases and 646 controls to estimate the AUC of *qXR* with 80% power, an alpha of 0.05 and a 5% margin of error^10–12^.

### Identification of cases and controls

Paper laboratory records were used to identify patients that had received a microbiological test for PTB (acid fact bacilli (AFB) smear, Xpert MTB/RIF or *Mycobacterium tuberculosis* (MTB) culture) at Kasturba Hospital from January 1^st^, 2017 – December 31^st^, 2017. Patients were eligible for inclusion if the specimen type tested was sputum, bronchoalveolar lavage, endotracheal aspirate or lung biopsy. Cases were defined as adults (≥18) diagnosed with PTB microbiologically confirmed by smear, Xpert MTB/RIF or culture (Mycobacteria Growth Indicator Tube, MGIT). Controls were defined as adults (≥18) who tested negative for PTB by smear and culture and who did not receive empiric treatment for PTB. All information was collected anonymously and patients were identified through a study identification number.

### Additional data sources

Electronic laboratory records were used to double check the microbiological status of the cases and controls and obtain the date of the specimen collection for the microbiological test. Radiological records were reviewed and Digital Imaging and Communications in Medicine (DICOM) files of posterior anterior (PA) CXR were extracted for cases and controls. In the case where multiple CXR were available for a single patient, the CXR taken on the date closest to microbiological test was selected. If a PA CXR was not available, a portable CXR was selected. Anterior posterior (AP) CXR were not otherwise selected. Patients without a CXR on file or whose CXR was completely unreadable (e.g., extremely blurry) were excluded. The date of the CXR was recorded for cases and controls. Electronic discharge summaries were reviewed to extract the following patient information: age, sex, HIV status, diabetes status, history of past TB, primary pulmonary diagnosis (e.g. lung-related or respiratory diagnosis) and empiric treatment initiation status. If no pulmonary diagnosis was indicated in the discharge summary, the primary non-pulmonary diagnosis was recorded.

### Chest radiograph interpretation

All CXR DICOM files for cases and controls were randomly assigned to one of two readers. Both radiologists were blinded to all clinical information and microbiological test results but were not blinded to the study hypothesis. The radiologists evaluated the CXR for the tags described in Table 1. Reader A was the head of the radiology department at Kasturba Hospital and had 20 years of experience. Reader B was a radiologist at Kasturba Hospital and had 5 years of experience. Anonymized CXR were then digitally sent to Qure.ai for analysis with *qXR* Version 2. Subsequently, each CXR was analyzed by *qXR* to determine the probability scores for the tags described in Table 1. The results from the *qXR* analysis were sent back to the authors via email in an Excel file.

**Table 1:** Definition of tags evaluated by qXR.

	Definition
Abnormal	Any abnormality on the CXR, including but not limited to those listed below. Borderline abnormalities marked as abnormal (i.e., any CXR that would NOT be reported outright as ‘Normal CXR’ or ‘No abnormality detected’)
Blunted costophrenic angle	Costophrenic angle blunted or obscured, could be due to effusion or pleural fibrosis
Cardiomegaly	Cardiothoracic ratio >0.5
Hilar Lymphadenopathy	Enlarged or prominent hilum, including hilar lymphadenopathy
Opacity	Any abnormal focal or generalized opacity or opacities in lung fields (blanket tag including but not limited to consolidation, cavity, fibrosis, mass, infiltrate, nodule, calcification, interstitial thickening etc.)
Cavity	Cavity
Consolidation	Pulmonary consolidation
Fibrosis	Any abnormal pulmonary fibrosis including interstitial fibrosis, fibrosis as part of fibrocavitatory lesion, etc.
Pleural Effusion	Pleural effusion
Tuberculosis Screen	Any sign suggesting pulmonary or extrapulmonary TB

### Inter-rater reliability

A consecutive sample of CXR from 30 microbiologically-confirmed positive patients and 30 microbiologically-confirmed negative patients who were tested for PTB in 2018 (distinct from the validation set) were used to assess the inter-rater reliability of Reader A and Reader B. Each reader analyzed all 60 CXR independently and blinded to the microbiological report and all other clinical information.

### Statistical methods

All data analysis was performed using R version 3.5.3. Kappa coefficients, and associated 95% confidence intervals (CI), were used to investigate inter-rater reliability of the radiologists for detection of specific chest abnormalities. The following scale was used for the interpretation of kappa coefficients: <0, poor; 0–0.20, slight; 0.21–0.40, fair; 0.41–0.60, moderate; 0.61–0.80, substantial; and 0.81–1.00, almost perfect^13^. To assess overall accuracy of *qXR*, receiver operating characteristic (ROC) analysis was used to determine the AUC, along with 95% CI. We did not pre-specify a threshold at which to assess accuracy measures for *qXR*. Instead, we assessed them using a range of thresholds higher than 0.5 (the default threshold of use suggested by Qure.ai for PTB screening) (personal communication, Preetham Putha, Qure.ai, 2019). Sensitivity and specificity were also calculated using Youden’s index, the threshold that simultaneously maximizes sensitivity and specificity^14^. No subgroup analyses were specified *a priori*. The same accuracy measures were also used to assess the validity of radiologists. In all instances where sensitivity, specificity, positive predictive value and negative predictive value were calculated, microbiological confirmation was used as the reference standard. To assess the accuracy of *qXR* for detection of specific chest abnormalities, ROC curves were generated and AUC was calculated, using radiologists’ interpretations as the reference standard.

## Results

### Participant inclusion

**Figure 1:**
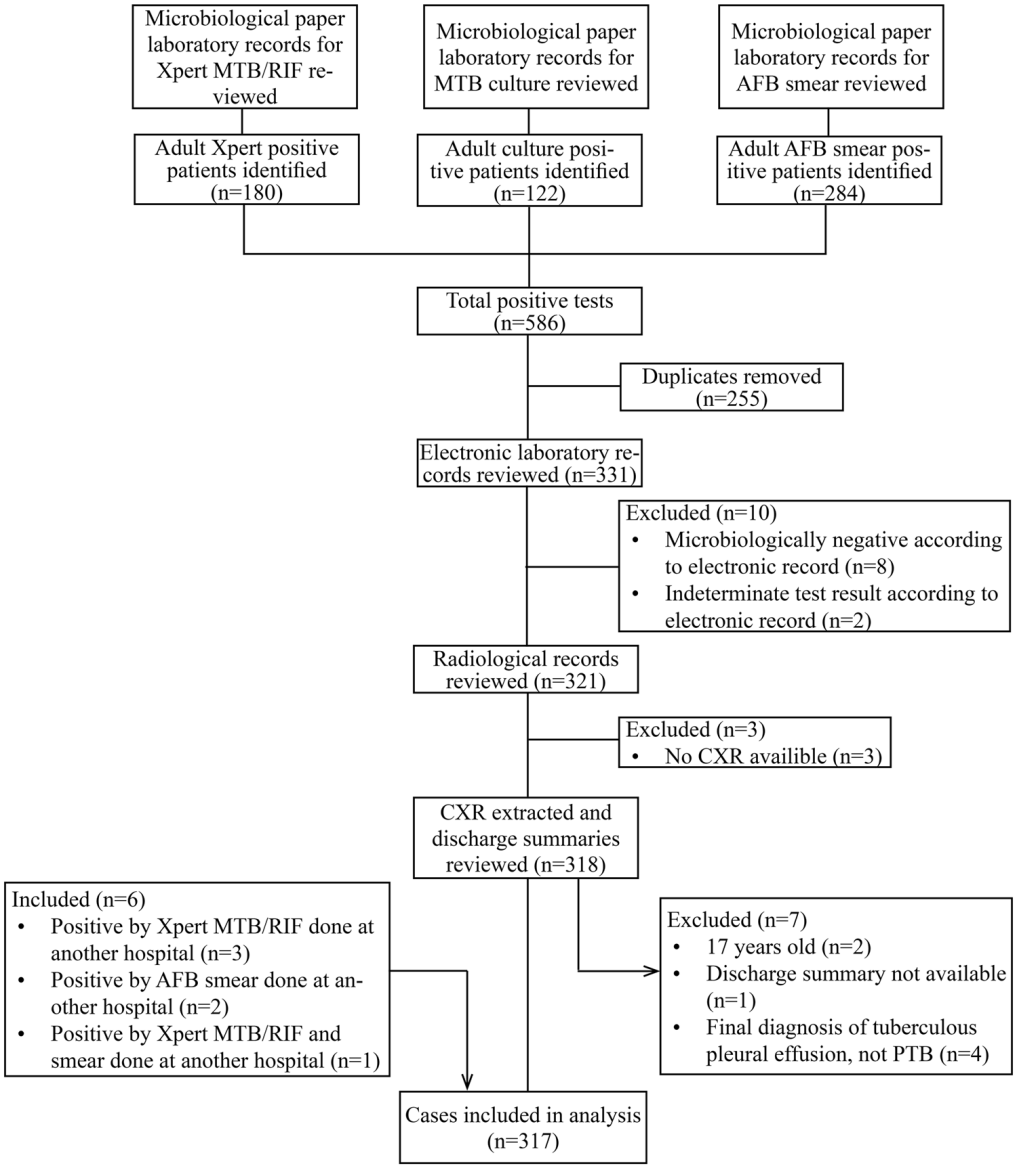
Flow chart for selection of cases. MTB: Mycobacterium tuberculosis; RIF: rifampicin; AFB: acid fast bacilli; CXR: chest radiography; PTB: pulmonary tuberculosis.

After reviewing the 2017 laboratory records for AFB smear, Xpert MTB/RIF and MTB culture test results, 331 individuals meeting the case definition were identified (Fig. 1). After reviewing electronic laboratory records, radiological records, and discharge summaries, 20 (6%) patients were excluded from the analysis for the following reasons: microbiologically negative according to electronic record (n = 8), indeterminate test result according to electronic record (n = 2), no CXR available (n = 3), under 18 years old (n = 2), discharge summary not available (n = 1) and final diagnosis of tuberculous pleural effusion (n = 4). Additionally, six controls were reclassified as cases after determining that they had microbiologically-confirmed PTB diagnosed at another hospital. Ultimately, 317 (96%) of the eligible cases were included in the analysis.

**Figure 2:**
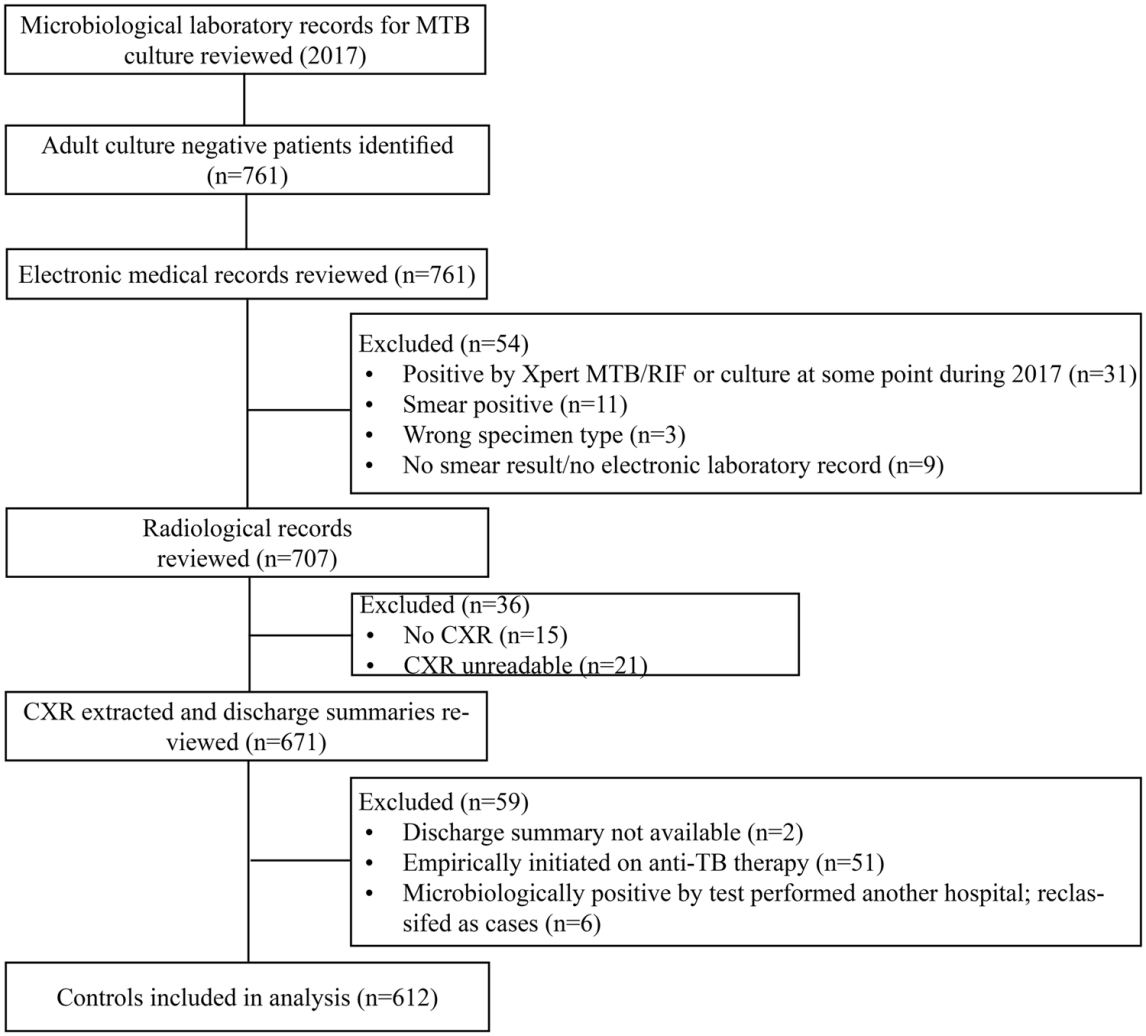
Flow chart for selection of controls. MTB: Mycobacterium tuberculosis; RIF: rifampicin; CXR: chest radiography; TB: tuberculosis.

After reviewing the 2017 laboratory records for MTB culture test results, 761 patients with negative cultures were identified (Fig. 2). After reviewing electronic laboratory records, radiological records and discharge summaries, 149 (20%) patients were excluded from the analysis for the following reasons: positive by Xpert MTB/RIF or culture at some point during 2017 according to electronic laboratory record (n = 31), smear positive (n = 11), wrong specimen type (n = 3), no smear result/no electronic record (n = 9), no CXR (n = 15), CXR unreadable (n = 21), discharge summaries were not available for review (n = 2) and empirically initiated on anti-TB therapy (n = 51). As previously mentioned, six controls were reclassified as cases. Ultimately, 612 (80%) of the eligible controls were included in the analysis.

### Participant characteristics

Among the 317 microbiologically-confirmed PTB cases included in the analysis, 237 (75%) were male and 80 (25%) were female. The mean age [SD] was 47 [16] years old. Over one-third of the cases, 113 (36%) had type 2 diabetes, 59 (19%) had a past history of PTB and 20 (6%) were HIV-positive (Table 2). Among the 317 PTB cases, 219 (69%) tested positive by AFB smear microscopy, 165 (52%) tested positive by Xpert MTB/RIF and 111 (35%) tested positive by culture. Furthermore, 189 (60%) tested positive by either Xpert MTB/RIF or culture and the remaining 128 (40%) tested positive by AFB smear alone (Table 3). Among cases, the median duration between specimen collection for the microbiological test and CXR was two days (Table 2).

**Table 2:** Baseline characteristics among cases and controls.

	Controls (N = 612)	Cases (N = 317)
**Age**		
Mean (SD)	54.4 (14.5)	47.0 (16.0)
**Gender, n (%)**		
Male	410 (67.0%)	237 (74.8%)
Female	202 (33.0%)	80 (25.2%)
**Comorbidities, n (%)**		
Type 2 Diabetes	164 (26.8%)	113 (35.6%)
Past History of PTB	79 (12.9%)	59 (18.6%)
HIV	27 (4.4%)	20 (6.3%)
**Days between Specimen Collection and CXR, n (%)**		
Mean (SD)	5.2 ± 19.8	5.1 ± 16.9
Median (IQR)	2.0 (1.0, 4.0)	2.0 (1.0, 3.0)

**Table 3:** Microbiological methods of diagnosis among pulmonary tuberculosis cases.

	Cases (N = 317) n (%)
**Diagnostic Methods**	
AFB Smear	219 (69.09%)
Xpert MTB/RIF	165 (52.05%)
Culture	111 (35.02%)
Xpert MTB/RIF or Culture	189 (59.62%)
AFB Smear Alone	128 (40.38%)

Note: The rows for individual tests (AFB Smear, Xpert MTB/RIF, Culture) refer to the percentage of patients who received that test. The patients may have also received additional diagnostic tests. The row for ‘Xpert MTB/RIF or Culture’ refers to the percentage of patients who had *at least* received Xpert MTB/RIF *or* Culture. The row for ‘AFB Smear Alone’ refers to patients who *only* received a smear and no other tests.

Among the 612 culture- and AFB smear-negative controls included in the analysis, 410 (67%) were male and 202 (33%) were female. The mean age [SD] was 54 [14.5] years old. Over one quarter of the controls, 164 (27%) had type 2 diabetes, 79 (13%) had a past history of PTB and 27 (4%) were HIV-positive (Table 2). Among the 612 controls, 534 (87%) had pulmonary conditions other than PTB and 78 (13%) had non-pulmonary conditions. The most common diagnoses were pneumonia (n = 159, 26%) and lung malignancies, masses and metastases (n = 94, 15%) (Table 4). Among controls, the median duration between specimen collection for the microbiological test and CXR was two days (Table 2).

**Table 4:** Pulmonary and non-pulmonary diagnoses among controls.

	Controls (N = 612) n (%)
**Pulmonary**	
Pneumonia	159 (25.98%)
Lung Malignancy/Mass/Metastasis	94 (15.36%)
Pleural Diseases (not PTB)	87 (14.22%)
Bronchiectasis	54 (8.82%)
Chronic Obstructive Pulmonary Disease	35 (5.72%)
Interstitial Lung Disease	26 (4.25%)
Aspergillosis	16 (2.61%)
Bronchial Asthma	13 (2.12%)
Nontuberculous Mycobacterial Pulmonary Infection	5 (0.82%)
Other	45 (7.35%)
**Non-Pulmonary**	
Malignancies	22 (3.59%)
Acquired Immunodeficiency Syndrome	15 (2.45%)
Sepsis	7 (1.14%)
Other	34 (5.56%)

PTB: pulmonary tuberculosis. Note: Pleural diseases include pleural effusion, empyema, pyopneumothorax, pneumothorax, hydropneumothorax.

### Inter-rater reliability of radiologists

Inter-rater reliability between Reader A and Reader B for detection of the abnormalities listed in Table 1 was assessed using a pilot set of 60 CXR (distinct from the validation set). Agreement for classification of a CXR as ‘TB Screen positive’ or ‘TB Screen negative’ was almost perfect (k = 0.83 [95% CI: 0.70, 0.97]). Agreement for detection of ‘fibrosis’ was almost perfect (k = 0.82 [95% CI: 0.67, 0.97]). Agreement for detection of ‘cavity’ and ‘cardiomegaly’ was substantial (k = 0.79 [95% CI: 0.62, 0.97] and k = 0.74 [95% CI: 0.46, 1.03]). Agreement for detection of ‘blunted costophrenic angle’, ‘pleural effusion’ and ‘opacity’ was moderate (k = 0.56 [95% CI: 0.34, 0.78], k = 0.52 [95% CI: 0.27, 0.78] and k = 0.48 [95% CI 0.21, 0.76]). Agreement for detection of ‘hilar lymphadenopathy’ and ‘consolidation’ was fair (k = 0.38 [95% CI: 0.06, 0.69] and k = 0.31 [95% CI: 0.08, 0.54]). Agreement for classification of a CXR as ‘abnormal’ was moderate (k = 0.45 [95% CI: 0.12, 0.78]) (Table 5).

**Table 5:** Inter-rater reliability of radiologists in pilot study (N = 60).

	Kappa Statistic	95% Confidence Interval	Level of Agreement
Abnormal	0.45	(0.12, 0.78)	Moderate
Blunted Costophrenic Angle	0.56	(0.34, 0.78)	Moderate
Cardiomegaly	0.74	(0.46, 1.03)	Substantial
Hilar Lymphadenopathy	0.38	(0.06, 0.69)	Fair
Opacity	0.48	(0.21, 0.76)	Moderate
Cavity	0.79	(0.62, 0.97)	Substantial
Consolidation	0.31	(0.08, 0.54)	Fair
Fibrosis	0.82	(0.67, 0.97)	Almost Perfect
Pleural Effusion	0.52	(0.27, 0.78)	Moderate
Tuberculosis Screen	0.83	(0.70, 0.97)	Almost Perfect

### Performance of qXR for detection of microbiologically-confirmed PTB

**Figure 3:**
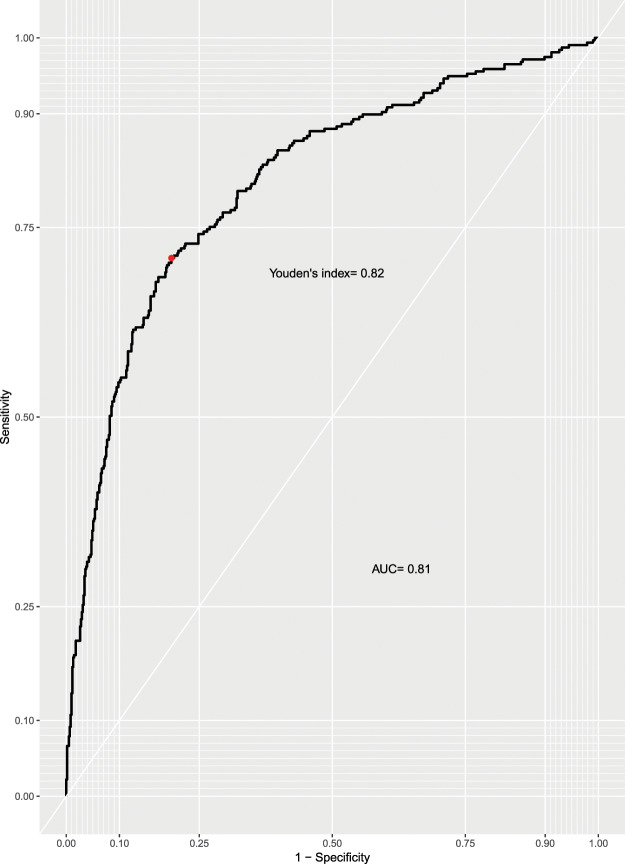
Performance of qXR for detection of microbiologically-confirmed PTB. AUC: area under the curve.

The pretest probability of PTB in our study was 34%. The AUC achieved by *qXR* for detecting microbiologically-confirmed PTB was 0.81 (95% CI: 0.78, 0.84) (Fig. 3). The threshold that maximized the sensitivity and specificity of *qXR* simultaneously was 0.818. Using a threshold of 0.818, *qXR* achieved a sensitivity of 71% (95% CI: 66%, 76%) and a specificity of 80% (95% CI: 77%, 83%) (Table 6).

**Table 6:** Sensitivity and Specificity of qXR at different thresholds.

Threshold	Sensitivity (95% CI)	Specificity (95% CI)
0.5	0.91 (0.88, 0.95)	0.35 (0.31, 0.38)
0.6	0.89 (0.85, 0.92)	0.46 (0.42, 0.50)
0.7	0.84 (0.80, 0.88)	0.61 (0.58, 0.65)
0.8	0.73 (0.68, 0.78)	0.76 (0.73, 0.79)
0.818*	0.71 (0.66, 0.76)	0.80 (0.77, 0.83)
0.9	0.64 (0.59, 0.69)	0.84 (0.82, 0.87)

^*^Youden’s index.

Comparatively, radiologists achieved a sensitivity of 56% (95% CI: 50%, 62%) and a specificity of 80% (95% CI: 77%, 83%). Sensitivity increased from 56% to 58% (95% CI: 51%, 65%) when the case definition was restricted to only include culture or Xpert MTB/RIF positive patients (Table 7). Using the classification of CXR as ‘abnormal’ as opposed to ‘TB Screen positive’, radiologists achieved a sensitivity of 94% (95% CI: 91%, 97%) and a specificity of 21% (95% CI: 18%, 24%) (Table 7).

**Table 7:** Validity of radiologists using different tags and different reference standards.

	Situation A	Situation B	Situation C
Sensitivity (95% CI)	0.56 (0.50, 0.62)	0.58 (0.51, 0.65)	0.94 (0.91, 0.97)
Specificity (95% CI)	0.80 (0.77, 0.83)	0.80 (0.77, 0.83)	0.21 (0.18, 0.24)
Positive Predictive Value (95% CI)	0.59 (0.53, 0.65)	0.47 (0.41, 0.54)	0.38 (0.35, 0.42)
Negative Predictive Value (95% CI)	0.78 (0.74, 0.81)	0.86 (0.83, 0.89)	0.88 (0.81, 0.92)

**Situation A:** ‘TB screen’ tag compared to microbiological reference standard of smear, culture or GeneXpert. **Situation B:** ‘TB screen’ tag compared to microbiological reference standard of culture or GeneXpert. **Situation C:** ‘Abnormal’ tag compared to microbiological reference standard of smear, culture or GeneXpert.

### Performance of qXR for detection of specific chest abnormalities

**Figure 4:**
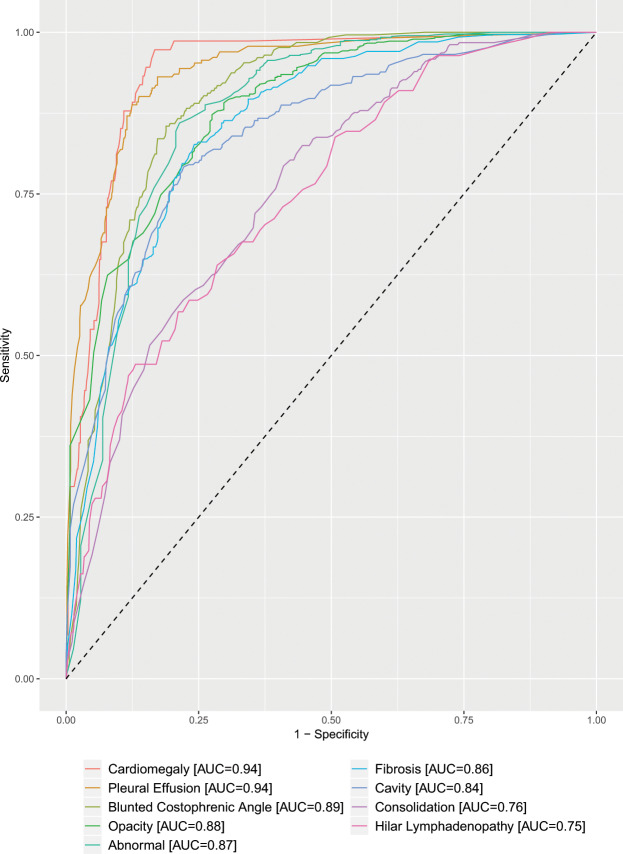
Performance of qXR for detection of specific chest abnormalities using radiologists’ interpretations as the reference standard. AUC: area under the curve.

The performance of *qXR* for detection of specific abnormalities was evaluated using the radiologist’s interpretation as the reference standard. The lowest AUC achieved by qXR, 0.75 (95% CI: 0.70, 0.80) and 0.76 (95% CI: 0.73, 0.79), were for detection of ‘hilar lymphadenopathy’ and ‘consolidation’, respectively. For detecting abnormalities ‘cavity,’ ‘fibrosis,’ ‘pleural effusion’, ‘opacity’ and ‘blunted costophrenic angle’, *qXR* achieved AUC ranging from 0.84 to 0.94. The highest AUC achieved by *qXR*, 0.94 (95% CI: 0.91, 0.96), was for detection of ‘cardiomegaly’. For the general classification of a CXR as ‘abnormal’, *qXR* achieved an AUC of 0.87 (95% CI: 0.84, 0.91) (Fig. 4).

## Discussion

In a tertiary care hospital, *qXR* demonstrated moderate sensitivity and specificity for the detection of PTB. Overall, we found *qXR* achieved a modest AUC of 0.81 (95% CI: 0.78, 0.84) for detection of PTB among patients with presumptive PTB in a tertiary care setting. This falls within the range of what has previously been reported by independent studies for CAD4TB (Delft Imaging Systems, Veenendaal, the Netherlands), the other commercially available CAD solution for PTB detection^15^. In a recent study funded and conducted by the developer of CAD4TB, the latest version of the software (Version 6) was found to have an AUC of 0.89 for the detection of PTB confirmed with Xpert MTB/RIF^11^.

Using Youden’s index (0.82), *qXR* achieved a sensitivity of 71% and specificity of 80% for detection of microbiologically-confirmed PTB. The target-product profile for a triage or referral test for PTB stipulates the minimum required sensitivity and specificity of the test must be 90% and 70%, respectively^16^. Our analysis shows that *qXR* does not meet these requirements simultaneously in a tertiary care setting. At a sensitivity of 90% *qXR* achieved a corresponding specificity of 42% (95% CI: 30%, 57%) and at a specificity of 70% *qXR* achieved a corresponding sensitivity of 77% (95% CI: 72%, 82%).

There are likely several factors contributing to the low specificity of *qXR*. One factor may have been the high prevalence of pulmonary conditions among the control group which are known to cause radiological manifestations similar to those caused by PTB. Over 85% of the control group was diagnosed with pulmonary or respiratory conditions and many likely had multiple concurrent lung conditions. Several of these conditions, namely pneumonia, lung cancer and aspergillosis, have been shown to mimic PTB on CXR^17–19^. Over 40% of the control population had either pneumonia or lung cancer. Another factor may have been the prevalence of past PTB among the controls. Past PTB can also present with persisting radiographic abnormalities that may be confused with current infection and disease^20^. When patients with a known history of past PTB were removed from the control group, the AUC increased from 0.81 to 0.83 (Supplementary Fig. 3). It’s likely a greater proportion of the controls had past PTB than what was reported in the patient discharge summaries.

The accuracy of *qXR* for differentiating normal from abnormal CXR and detecting specific chest abnormalities was assessed using a radiologist’s interpretation as the reference standard. The software achieved an AUC of 0.87 (95% CI: 0.84, 0.91) for differentiating normal from abnormal CXR. This is lower than the AUC reported by Qure.ai of 0.93^21^. The AUC for detection of individual abnormalities ranged from 0.75 to 0.94. Compared to the results reported by Qure.ai, our study showed similar, often slightly lower, AUC for the detection of individual abnormalities^21^. The abnormalities that had the lowest inter-rater agreement between the two radiologists (‘consolidation’ and ‘hilar lymphadenopathy’) also had the lowest AUC.

### Strengths and limitations

This study has several strengths. Both the radiologists and Qure.ai were blinded to the microbiological test results and other clinical information when reviewing the CXR of participants. As such, the risk of interpretation (or review) bias is low. Exclusion of study participants was limited (6% of eligible cases and 20% of eligible controls) and all reasons for exclusions were reported. Discharge summaries were reviewed for all patients to confirm diagnoses and empirically treated patients were removed from the control group.

Another strength of our study is that the protocol was designed and the data analysis was performed independently from Qure.ai. Furthermore, the study population did not contribute any CXR to the development and training process of *qXR* as this may have artificially enhanced the software’s validity.

One of the primary limitations of this study is the use of the case-control design. As a result of the study design, the applicability of our results is limited to tertiary care settings in high TB-burden, low HIV prevalence settings and cannot be generalized to other contexts where the spectrum of diseased and non-diseased patients may differ. Moreover, all empirically treated patients (n = 51) were excluded from analyses so the study population is not fully representative of clinical practice.

Another source of bias is the use of an imperfect reference standard. In our study, 40% of cases were diagnosed by smear alone therefore there is risk of disease misclassification. However, we estimate that the risk is minimal given that patient discharge summaries were reviewed in addition to paper and electronic laboratory records. Furthermore, there was only a small change in AUC from 0.81 to 0.79 when patients diagnosed by smear alone were excluded from the analysis (Supplementary Fig. 2).

Another limitation of our study is that CXR were only read by one radiologist as opposed to multiple radiologists or a panel of radiologists. As a result, there may have been some degree of misclassification of the presence of chest abnormalities.

## Conclusions

The focus of this study was assessing the accuracy of *qXR* in a tertiary care setting with a high pretest probability of PTB. Because many patients who present at tertiary hospitals in India have symptoms suggestive of PTB, such as weight loss and cough, accurate and rapid triage tests that can rule out the disease are needed^2^.

Our study demonstrates that *qXR* can detect PTB with modest accuracy. The software is threshold dependent and at a threshold that achieves the level of sensitivity required for a ‘rule out’ test, specificity was low. Our study suggests CAD software might have limited specificity for PTB detection in tertiary care settings because of the high prevalence of pulmonary conditions that cause lung shadows. Accuracy in detection of many individual chest abnormalities, including those specific for PTB such as cavity, was relatively high.

There is likely a larger role for CAD software as a triage test for PTB at the primary care level in settings where access to radiologists is limited, compared to at the tertiary care level. At the primary care level, CAD could be rolled out faster and more broadly than human readers, increasing capacity for PTB screening. This is especially relevant in the Indian context where the burden of PTB is high and the ratio of radiologists to people is low^7^. However, further field studies and implementation research are needed to understand how this technology will work in real-world conditions. Another potential role for CAD that needs to be explored is in assisted reading of CXR to help human readers, especially non-experts, in interpreting CXR.

Further research is necessary to assess the software’s accuracy in other populations and in the screening use-case. Larger prospective studies that can better assess heterogeneity in important subgroups such as HIV and smear status are needed.

As accuracy is established for both the screening and triage use-cases with pre-specified thresholds, field studies that assess the software’s accuracy and cost-effectiveness in real-world conditions will also be needed. Subsequently, the value added of CAD in PTB triage and screening algorithms can be established and policy guidelines can be developed.

### Ethics approval and consent to participate

This study was reviewed and approved by the central ethics committees in Manipal University, India (Manipal Academy of Higher Education Ethics Committee Number 006/2018) and McGill University, Canada (Institutional Review Board Study Number A06-E48-18B). The need for written informed consent was waived by both the Manipal Ethics Committee and the McGill Institutional Review Board as all data sources used (laboratory records, patient discharge summaries and CXR) were previously available and no patients needed to be contacted. Additionally, all information was collected anonymously and patients were identified through a study identification number. The study was performed in accordance with ICH GCP guidelines and regulations.

### Supplementary information

Supplementary figures

## Data Availability

The datasets used and/or analysed during the current study are available from the corresponding author on reasonable request.
