# Author Correction: Deep learning, computer-aided radiography reading for tuberculosis: a diagnostic accuracy study from a tertiary hospital in India

**DOI:** 10.1038/s41598-024-57763-y

**Published:** 2024-03-26

**Authors:** Madlen Nash, Rajagopal Kadavigere, Jasbon Andrade, Cynthia Amrutha Sukumar, Kiran Chawla, Vishnu Prasad Shenoy, Tripti Pande, Sophie Huddart, Madhukar Pai, Kavitha Saravu

**Affiliations:** 1 Department of Epidemiology, Biostatistics and Occupational Health, McGill University, Montreal, Canada; 2 McGill International TB Centre, McGill University, Montreal, Canada; 3 Department of Radiodiagnosis, Kasturba Medical College, Manipal, Manipal Academy of Higher Education, Manipal, India; 4 Department of Medicine, Kasturba Medical College, Manipal, Manipal Academy of Higher Education, Manipal, India; 5 Department of Microbiology, Kasturba Medical College, Manipal, Manipal Academy of Higher Education, Manipal, India; 6 Department of Infectious Diseases, Kasturba Medical College, Manipal, Manipal Academy of Higher Education, Manipal, India; 7 Manipal McGill Program for Infectious Diseases, Manipal Centre for Infectious Diseases, Prasanna School of Public Health, Manipal Academy of Higher Education, Manipal, India

Correction to: *Scientific Reports* 10.1038/s41598-019-56589-3, published online 14 January 2020

This Article contains errors in the data availability section, where the conditions for sharing the data have been omitted.

The corrected data availability statement reads:

The data supporting the findings of this study are not publicly available due to statutory restrictions on sharing patient/subject data under Indian Privacy Laws. The data are, however, available from the corresponding author upon reasonable request and under the condition to sign a non-disclosure and confidentiality agreement that includes the reasonable restrictions of (a) providing the data on an anonymous basis, (b) utilising the data only for research, (c) no commercial exploitation.

