# Supplementary material for: Deep learning, computer-aided radiography reading for tuberculosis: a diagnostic accuracy study from a tertiary hospital in India
Source: Sci Rep. 2020 Jan 14;10:210. doi: 10.1038/s41598-019-56589-3 (PMC6959311; doi:10.1038/s41598-019-56589-3)
Supplement: Supplementary file 1 — Supplementary figures [file 41598_2019_56589_MOESM1_ESM.docx]

**Deep learning, computer-aided radiography reading for tuberculosis: a diagnostic accuracy study from a tertiary hospital in India**

Madlen Nash^1,2^, Rajagopal Kadavigere^3^, Jasbon Andrade^3^, Cynthia Amrutha Sukumar^4^, Kiran Chawla^5^, Vishnu Prasad Shenoy^5^, Tripti Pande^2^, Sophie Huddart^1,2^, Madhukar Pai*^1,2,7^, Kavitha Saravu*^6,7^.

*Contributed equally

1 Department of Epidemiology, Biostatistics and Occupational Health, McGill University, Montreal, Canada

2 McGill International TB Centre, McGill University, Montreal, Canada

3 Department of Radiodiagnosis, Kasturba Medical College, Manipal, Manipal Academy of Higher Education, Manipal, India

4 Department of Medicine, Kasturba Medical College, Manipal, Manipal Academy of Higher Education, Manipal, India

5 Department of Microbiology, Kasturba Medical College, Manipal, Manipal Academy of Higher Education, Manipal, India

6 Department of Infectious Diseases, Kasturba Medical College, Manipal, Manipal Academy of Higher Education, Manipal, India

7 Manipal McGill Program for Infectious Diseases, Manipal Centre for Infectious Diseases, Prasanna School of Public Health, Manipal Academy of Higher Education, Manipal, India

**Author Emails:**

[madlen.nash@mail.mcgill.ca](mailto:madlen.nash@mail.mcgill.ca)

[rajarad@gmail.com](mailto:rajarad@gmail.com)

[jasbon.andrade@gmail.com](mailto:jasbon.andrade@gmail.com)

[cynthiaamrutha@gmail.com](mailto:cynthiaamrutha@gmail.com)

[kiran.chawla@manipal.edu](mailto:kiran.chawla@manipal.edu)

[vishnumanav@gmail.com](mailto:vishnumanav@gmail.com)

[tripti.pande@mail.mcgill.ca](mailto:tripti.pande@mail.mcgill.ca)

[sophie.huddart@mail.mcgill.ca](mailto:sophie.huddart@mail.mcgill.ca)

[madhukar.pai@mcgill.ca](mailto:madhukar.pai@mcgill.ca)

[kavithasaravu@gmail.com](mailto:kavithasaravu@gmail.com)

**Corresponding author:**

Kavitha Saravu, MD, DNB, DTM&H

Professor & Head, Dept of Infectious Diseases, KMC, Manipal

Coordinator, Manipal Center for Infectious Diseases

Manipal Academy of Higher Education, Manipal, Karnataka, India

Email: [kavithasaravu@gmail.com](mailto:kavithasaravu@gmail.com); [kavitha.saravu@manipal.edu](mailto:kavitha.saravu@manipal.edu)

Among patients with type 2 diabetes, *qXR* achieved an AUC of 0.81 (95% CI: 0.76, 0.86) (Supplemental Figure 1). Restricting the case definition to patients who tested positive for PTB by either Xpert MTB/RIF or culture, resulted in an AUC of 0.79 (95% CI: 0.75, 0.83) (Supplemental Figure 2). Restricting the control definition to exclude patients with a past history of PTB resulted in an AUC of 0.83 (95% CI: 0.80, 0.86) (Supplemental Figure 3). Restricting the maximum duration between the date of specimen collection (for the microbiological test) and the date the CXR was taken to be less than or equal to 60 days, resulted in an AUC of 0.82 (95% CI: 0.78, 0.86) (Supplemental Figure 3).

**Supplementary Figure 1. Performance of qXR for detection of microbiologically-confirmed PTB among individuals with type 2 diabetes.** AUC: area under the curve.

**Supplementary Figure 2. Performance of qXR for detection of microbiologically-confirmed PTB with the case group restricted to those diagnosed by either Xpert MTB/RIF or culture.** AUC: area under the curve.

**Supplementary Figure 3. Performance of qXR for detection of microbiologically-confirmed PTB when the control group has no patients with past history of PTB.** AUC: area under the curve.

**Supplementary Figure 4. Performance of qXR for detection of microbiologically-confirmed PTB with less than or equal to 60 days between specimen collection for microbiological test and chest radiograph.** AUC: area under the curve.
